# Microglia as hackers of the matrix: sculpting synapses and the extracellular space

**DOI:** 10.1038/s41423-021-00751-3

**Published:** 2021-08-19

**Authors:** Joshua D. Crapser, Miguel A. Arreola, Kate I. Tsourmas, Kim N. Green

**Affiliations:** Department of Neurobiology and Behavior, University of California, Irvine, CA USA

**Keywords:** Neuroscience, Perineuronal nets, Microglia, Extracellular matrix, Neuroinflammation, Immunology, Cell biology

## Abstract

Microglia shape the synaptic environment in health and disease, but synapses do not exist in a vacuum. Instead, pre- and postsynaptic terminals are surrounded by extracellular matrix (ECM), which together with glia comprise the four elements of the contemporary tetrapartite synapse model. While research in this area is still just beginning, accumulating evidence points toward a novel role for microglia in regulating the ECM during normal brain homeostasis, and such processes may, in turn, become dysfunctional in disease. As it relates to synapses, microglia are reported to modify the perisynaptic matrix, which is the diffuse matrix that surrounds dendritic and axonal terminals, as well as perineuronal nets (PNNs), specialized reticular formations of compact ECM that enwrap neuronal subsets and stabilize proximal synapses. The interconnected relationship between synapses and the ECM in which they are embedded suggests that alterations in one structure necessarily affect the dynamics of the other, and microglia may need to sculpt the matrix to modify the synapses within. Here, we provide an overview of the microglial regulation of synapses, perisynaptic matrix, and PNNs, propose candidate mechanisms by which these structures may be modified, and present the implications of such modifications in normal brain homeostasis and in disease.

## A small cell with many hats: the emerging complexity of microglia

Far from acting simply as a structural glue that holds neuronal networks together, as suggested by the Greek word from which the name “glia” is derived, it is now readily apparent that microglia and macroglia (astrocytes, oligodendrocytes) are important determinants of brain development and health [1]. Microglia in particular have been the focus of further reappraisal, as their functional repertoire has extended from the classically immune—detecting and resolving injury and invasive pathogens—to more non-immune roles in the homeostatic brain [2–4]. These findings have occurred across a backdrop of increasingly elegant methodological advances, including single-cell analyses [5–7], microglial ablation paradigms [8–12], and in vivo imaging techniques [13–16], that together have characterized the dynamic influence microglia have on virtually all major central nervous system (CNS) cell types over the lifespan of an organism. However, this increasing functional complexity suggests a greater opportunity for dysfunction and dyshomeostasis should microglia fail to properly perform their cellular roles at the appropriate times, as supported by a growing body of evidence implicating microglia as drivers of disease pathogenesis [17]. Therefore, taking stock of the homeostatic functions performed by these cells—at this critical juncture of glial biology research—may provide insight into what goes wrong in disease and how such deficits may be best targeted in the clinic.

Microglia and other resident tissue macrophages display considerable diversity across organs at the gene expression and chromatin levels [18], reflecting the varying developmental and functional roles they play in each tissue [19–22]. The recent wave of studies characterizing microglia at single-cell resolution indicate extensive transcriptional heterogeneity during development and disease, with a more homogeneous population evident in the homeostatic adult brain [5–7, 23, 24]. If an empty niche is available in the tissue myeloid compartment, cues from the local microenvironment can reprogram infiltrating bone marrow-derived monocytes or ontogenically foreign macrophages into microglia-like phenotypes [25–30]. The extent of transcriptional reprogramming appears to depend on the yolk-sac or hematopoietic origin of the cell in question [8, 26], at least in the CNS, where the adult resident macrophage population (e.g., microglia) derives from yolk-sac erythromyeloid progenitors [31–34]. The capacity for macrophage re-education, combined with the diverse transcriptional profiles of resident macrophages in different organs and stages of life, emphasizes the complexity of homeostatic functions performed by tissue myeloid cells, even in adults.

Research into the role of microglia in neurodevelopment has revealed numerous essential functions not directly related to immunity but essential for proper brain organization and tissue health [1, 21, 22]. Additional investigation into early microglial functions has drawn some to label these cells “architects” of the developing CNS [1, 35], with top-down roles in the spatial organization and survival of both neuronal and non-neuronal cell types. In the perinatal period, microglia orchestrate spatial patterning of neurons through a variety of regulatory mechanisms that dictate neuronal/neuronal precursor cell (NPC) survival, i.e., signaling through soluble/membrane-bound microglial factors (glutamate, reactive oxygen species, and TNF-α) that induce programmed cell death prior to microglial phagocytosis of debris, reported phagocytosis of live cells (“phagoptosis”), and secretion of pro-survival factors (e.g., insulin-like growth factor; IGF-1) [21]. Microglia continue to regulate neurogenesis in the adult brain by phagocytic maintenance of the hippocampal NPC pool [36]. A major re-evaluation of microglial effector functions in the brain was spurred by the discovery of synaptic engulfment and pruning by postnatal microglia that occurs in an activity- and complement-dependent manner to refine the neural circuitry [37–39] and continues to some extent in adults during memory processing [40].

Extensive work in recent years has elucidated the regulation of other glial cells by microglia, and vice versa, particularly in regard to astrocytes [41–44]. Microglia induce a neurotoxic phenotype in astrocytes (termed “A1” astrocytes) following LPS via release of microglial IL-1ɑ, TNF-ɑ, and C1q that promotes neuronal and oligodendrocyte death; loss of these factors (i.e., in *Il1ɑ*^−/−^*Tnf*^−/−^*C1qɑ*^−/−^ triple knockout mice) or microglia themselves (*Csf1r*^−/−^ mice) blocks A1 astrocyte formation [45]. Detrimental gain-of-function properties in neurotoxic astrocytes are accompanied by a loss of beneficial properties, including their support of neuronal outgrowth and synaptogenesis [45]. Subsequent studies determined that blocking these regulatory microglial factors by genetic or pharmacologic means ameliorates such neurotoxic astrocyte reactivity and/or neuronal death in models of amyotrophic lateral sclerosis (ALS) [46], Parkinson’s disease [47], optic nerve crush [48], glaucoma [48, 49], and aging [50]. It should be noted that the constitutive *Il1ɑ*^−/−^*Tnf*^−/−^*C1qɑ*^−/−^ triple knockout mice utilized in many of the aforementioned studies on astrocyte reactivity in disease will have off-target effects in a number of pathways, given the importance of these three molecules in a broad array of signaling cascades and biological functions [39, 51–53]. Therefore, the in vivo findings generated from these mice should be validated with targeted in vitro approaches, as in [45, 46], or with the in vivo use of specific pharmacological agents (e.g., neutralizing antibodies to IL-1ɑ, TNF-ɑ, and C1q [48], or the glucagon-like peptide-1 receptor agonist NLY01 [47, 49]) and/or conditionally inducible knockout models.

In addition to affecting oligodendrocyte number via the cytotoxic effects of reactive astrocytes [45], microglia also choreograph oligodendrocyte patterning directly in disease and under homeostatic conditions [2, 54]. Postnatal microglia are required for early oligodendrocyte precursor cell (OPC) maintenance and maturation and continue to support the OPC pool in adulthood [55], and minocycline-based inhibition of postnatal microglial activation impairs oligodendrocyte differentiation [56]. Furthermore, early microglia promote developmental myelinogenesis [55] at least in part through the production of myelinogenic IGF-1 by a CD11c^+^ microglial subpopulation [57], and myelin in the adult brain is phagocytosed by microglia, where it accumulates with age [58, 59]. Interactions with oligodendrocytes in disease are multifaceted, such that microglia can support remyelination in certain cases, as in the secretion of activin A to promote OPC proliferation and differentiation [60], and persistently disrupt OPC/oligodendrocyte population dynamics and myelination in others [61].

Thus, despite traditional classifications of microglia as immune cells first, mounting data indicate that they are intricately entwined in the complex process of brain tissue development and adult brain homeostasis, interacting with virtually every cell type in the brain to orchestrate non-immune as well as classically immune-related processes. Microglial cellular functionality may be perturbed in one of two ways: through (1) toxic gain-of-function, as exemplified by chronically activated microglia that fail to resolve ongoing proinflammatory cytokine and neurotoxin production (e.g., plaque-associated microglia in Alzheimer’s disease [62]), or (2) loss of beneficial or protective function. An example of the latter may be found in the failure to appropriately prune neuronal synapses in development, which may contribute to autism spectrum disorders [63–65]. The extent to which these changes contribute to brain disease depends on a multitude of contextual factors, including brain age, existing disease pathology, and disease-relevant genetic and environmental risk factors, among others.

To gain insight into the roles they play, microglia may be depleted via toxin-, genetic-, and pharmacological-based methods, and we have recently reviewed the comparative advantages, disadvantages, and caveats of these different approaches [11]. To serve as an illustration, our lab has previously developed pharmacological paradigms of microglial depletion with inhibitors of colony-stimulating factor 1 receptor (CSF1R), such as PLX3397 and PLX5622, signaling through which microglia are dependent for survival [10, 66–71]. Inhibitor-induced depletion of microglia occurs in the absence of behavioral deficits, cytokine storm, brain pathology, or replacement by peripheral myeloid cells [10] and appears to be indefinitely maintainable as long as inhibition is continued (at least 6 months) [71]. Such approaches have been utilized to study the effects that microglia exert on not only neuronal and synaptic functions in health and disease [66, 67, 72] but also on other glial cells. For instance, astrocyte reactivity is detected in Huntington’s disease (HD) [45], and we reported resolution of disease-associated astrogliosis with microglial depletion via CSF1R inhibition in HD model mice [73] that is similar to the observed effects of microglial depletion on astrocyte reactivity in the context of methotrexate chemotherapy [61], consistent with the increasingly studied function of microglia as regulators of astrocyte responses.

Restoring CSF1R signaling following inhibitor treatment by inhibitor withdrawal induces full repopulation of the microglial niche via proliferation of surviving microglia [10, 74], which may be exploited to correct dysfunctional microglial phenotypes by replacing the old with new cells. This aspect of microglial biology has been utilized to promote brain recovery after traumatic brain injury (TBI) [75], neuronal lesion [76], and aging [77] by resolving chronically activated or otherwise dyshomeostatic microglia in vivo. When discussing CSF1R-based microglial depletion models, it should be noted as an experimental caveat that CSF1R is also expressed by peripheral myeloid cells, as well as microglia, and accordingly, a growing number of studies are examining the peripheral effects of CSF1R inhibition [71, 78–84]. However, such peripheral off-target effects in studies focused on microglial function may be controlled by utilizing subtypes or doses of CSF1R inhibitors that achieve little to no blood-brain barrier (BBB) penetrance in healthy adult mice [11], such as PLX73086 [85], Ki20227 [86], or PLX3397 at 75 ppm [10]. Ultimately, by eliminating microglia and observing the consequences of their absence and/or renewal on brain physiology in homeostasis and disease and whether these processes normalize injury- or disease-associated deficits, we may make inferences about their function in the brain.

In this review, we will discuss recent findings regarding microglial functionality in the developing and adult brain, with a special focus on the more nebulous non-immune roles these cells serve and how such functions may go wrong in disease. Microglia exert top-down influence on all major components of the modernly conceptualized tetrapartite synapse [87, 88], namely, pre- and postsynaptic compartments [37, 89], glia [45, 55], and the extracellular matrix (ECM) [72, 73, 90, 91]. The microglial modulation of astrocytes and oligodendrocytes has been reviewed eloquently elsewhere [22, 41–44, 54]. We will focus here on the interactions between microglia and ECM compartments, primarily regarding how the former influence the latter, and how this may relate to synaptic remodeling. Where appropriate, we will draw from our expertise in microglial ablation techniques to showcase the unique efficacy of these tools in elucidating general principles of microglial biology and new ways in which they can be utilized to drive novel discoveries in this field.

## Microglia and the extracellular matrix in health and disease

Perhaps more aptly considered the ‘glue’ of the nervous system than glia themselves, the ECM is a highly complex and dynamic molecular meshwork with roles in plasticity, biophysical protection, and cell signaling [87, 92–94]. While its role as an extracellular scaffold is well known, less appreciated is the ability of the ECM to limit, and thus functionally compartmentalize, the diffusion and localization of key molecules [87], including neurotransmitters [95, 96], ions [97–99], and membrane receptors [100–102]. Furthermore, matrix molecules such as the abundant chondroitin sulfate proteoglycans (CSPGs), which consist of glycosaminoglycan side chains attached to a core protein, may inhibit neurite and axon growth [103–108], myelination and remyelination by oligodendrocytes/OPCs [109–111], and neural stem cell migration [112]. This has been classically illustrated in astrocytic glial scars that form after injury (e.g., spinal cord injury) and serve as a physicochemical barrier to neuronal recovery and axonal regrowth, thought to be due in large part to the consequent deposition of matrix components such as CSPGs into the interstitial ECM by reactive astrocytes [111, 113–116]. This interpretation is complicated by the discovery that certain glial scar components (such as specific CSPG subtypes [117, 118]), and the glial scar as a whole [119–121], both appear to facilitate axon regeneration and neurite outgrowth. Additionally, disruption of injury-induced astrocyte responses upstream of scar formation impedes recovery by impairing injury containment, BBB repair, and inflammation resolution [121–124], and astrocytes themselves may adopt multiple reactive phenotypes that likely exist on a functional spectrum [44, 125] and that may, in turn, be influenced by proteoglycans [123, 126]. Whatever new findings future studies may hold, it is clear that the ECM has a profound influence on brain health and disease.

As in the case of astrocyte-mediated scar formation, the ECM and interstitial environment critically regulate neuroinflammation and the immune response. Structural components of the ECM and products of its degradation may serve as damage-associated molecular patterns (DAMPs) that induce or suppress microglial reactivity by signaling through pattern recognition receptors (e.g., Toll-like receptors), as reviewed elsewhere [127–129]. For instance, culturing microglia on a CSPG substrate in vitro induces microglial activation, proliferation, and the expression of IGF-1, MMP-2, and MMP-9, whereas pharmacological inhibition of CSPG production with xyloside following spinal cord injury differentially alters inflammation and cytokine production depending on the timing of treatment [130]. Alternatively, disaccharides generated from the degradation of CSPGs with the bacterial enzyme chondroitinase ABC (ChABC) confer an activated noncytotoxic microglial phenotype that is associated with protection in experimental autoimmune encephalomyelitis [131], spinal cord injury [130], and multiple models of neurotoxicity [132, 133]. This observation may serve as a confounding factor in studies on the effects of ECM on microglia and inflammation that utilize ChABC, and therefore should be kept in mind when designing such experiments. In addition to the activation state, ECM components may also affect microglial adhesion, migration, and morphology, as in the case of cell-matrix signaling mediated by transmembrane ECM receptors known as integrins [134–136]. However, rather than the autonomous effects of ECM on neuroimmunology, in this review we will focus on the perspective of top-down microglial influences on the matrix and how these processes relate to changes in the synaptic landscape, while keeping in mind the bidirectional nature of ECM−immune interactions.

In terms of composition, the brain ECM is made up largely of proteoglycans, such as CSPGs and heparan sulfate proteoglycans (HSPGs), glycoproteins, such as laminins and tenascins, and glycosaminoglycans, such as the abundant hyaluronan [87, 93], among other molecules (e.g., collagens) that together constitute approximately 20% of the overall brain volume [96, 137]. The structural molecules that constitute the brain ECM and the proteases that are secreted to remodel it are produced by neurons, astrocytes, microglia, and oligodendrocyte lineage cells with varying degrees of overlap in terms of cellular origin depending on the molecule in question [94, 111, 138, 139]. The ECM can be partitioned into structural subtypes based on organization and composition, which generally include (1) the basement membrane of the BBB, (2) the diffuse ECM found in interstitial and perisynaptic spaces, (3) the condensed, reticular ECM that ensheathes neuronal subsets and their perisomatic synapses to form structures known as perineuronal nets (PNNs), and (4) the perinodal ECM that surrounds nodes of Ranvier within axons and that displays compositional resemblance to PNNs [94, 111]. Along with synaptic terminals and glial cells, the diffuse perisynaptic matrix and the synaptic ECM of PNNs constitute the fourth compartment and most recent addition to the conventional model of synaptic function, the tetrapartite synapse [87, 93], and recent studies report that both structures are dynamically regulated by microglia in the homeostatic adult brain [72, 73, 90, 91].

### Perineuronal nets

Discovered by Camillo Golgi and dismissed by Ramón y Cajal as a fixation artifact, perineuronal nets are specialized ECM structures that condense in a reticular fashion around the soma and proximal neurites (dendrites and axon initial segment) of neurons throughout the brain during development [94, 140]. Although PNNs are associated primarily with fast-spiking parvalbumin (PV)-expressing GABAergic interneurons, particularly in the cortex of the brain [141, 142], they are evident throughout the CNS and across a variety of neuronal subsets [92, 94, 142–145] and are generally (but not always) labeled with the lectin *Wisteria floribunda* agglutinin [94]. These formations serve as a molecular scaffold to stabilize and regulate the synapses they surround and reach adult levels during the closure of critical periods of neuroplasticity [92, 146], and the genetic or enzymatic removal of PNNs or their components are capable of reinstating critical period-like plasticity [146–149]. In line with their role as a synaptic scaffold, PNNs have been proposed as the molecular basis of long-term memory storage [150], and their experimental removal disrupts the consolidation [151, 152] and recall [152, 153] of various types of remote fear memories. Thus, the heightened plasticity afforded by PNN loss may impair long-term memory fidelity due to interference from new memory traces [92, 154, 155]. Physiologically, PNNs also protect host cells from neurotoxins such as Aβ_1-42_ and oxidative stress [156, 157], regulate neuronal excitability [158, 159], and augment neuronal firing by reducing membrane capacitance akin to myelin sheaths [160], thereby influencing excitatory-inhibitory balance. Furthermore, the removal of PNN components alters synaptic transmission [161], synaptic ion channel/neurotransmitter receptor localization [162], and synapse number [163, 164]. While future experiments will determine whether some of these effects at least partly result from changes to the perisynaptic matrix rather than the PNN proper, particularly in the case of brevican modifications [165], these findings taken together underscore the truly multimodal effects that the brain ECM in general, and PNNs specifically, exert on the cells they associate with and enwrap.

Previous studies have postulated that microglia may drive PNN loss in certain disease contexts due to their ability to secrete matrix-degrading enzymes (e.g., matrix metalloproteinases; MMPs) and/or their molecular activators or inhibitors [166–169]. Indeed, we have recently shown that CSF1R inhibitor-based microglial depletion prevents disease-associated PNN reductions in models of Huntington’s [73] and Alzheimer’s disease (AD) [90]. PNN components were evident in microglia in both AD mouse and human brain tissue, where they also colocalized with canonical dense-core plaques [90]. That we observed similar effects on PNN abundance in the relative absence of microglia across these models is striking, both due to their differential etiologies—intracellular vs. extracellular protein accumulation in the R6/2 HD [170] and 5xFAD model of AD [171], respectively—and the variable microglial phenotypes we observed, which resembled “classical activation” in 5xFAD [90] but not R6/2 brains [73], where they instead were associated with an interferon signature marked by enrichment of type I (IFNɑ, IFNβ) and type II (IFNγ) signaling pathways. In a similar vein, we extended these findings to *Csf1r*^+/−^ mice, a neurodegenerative model of adult-onset leukoencephalopathy with axonal spheroids and pigmented glia (ALSP) [172, 173], where early disease stage microglia that displayed homeostatic marker loss, but not proinflammatory gene upregulation, induced decreases in PNNs and presynaptic puncta (Arreola et al. [174]). In the same study, we confirmed these changes with inducible microglia-specific *Csf1r* haploinsufficiency (*Cx3cr1*^*+/CreERT2*^*: Csf1r*^*+/fl*^ mice) and their normalization following CSF1R inhibitor-based depletion, validating the microglial origin of ALSP PNN and synaptic deficits. In addition, a recent study reported that microglial depletion with the specific CSF1R inhibitor PLX5622 prevents PNN loss induced by ketamine or 60 Hz light entrainment [175]. Therefore, it appears that microglia drive PNN loss in disease, as has been suggested based on temporal analysis of microglial activation and their accumulation of net material in prion disease [166, 176, 177] and following infection with human or simian immunodeficiency virus (HIV or SIV), which preferentially infect microglia and cause PNN degradation [168, 178, 179].

PNN deficits/decreases have also been observed across more diverse diseases, many if not all of which are also generally associated with microglial activation, including multiple sclerosis [169], stroke [180–184], traumatic brain injury [185, 186], spinal cord injury [187], epilepsy [188, 189], obesogenic high fat and high sugar diet consumption [190], glioma [160], Alzheimer’s disease [90, 191–193], and schizophrenia [167, 194–196]. The loss of protective PNNs leaves PV^+^ and other enwrapped neurons susceptible to injury [92, 156, 157], and accordingly, we found that PNN reductions preceded decreases in PV^+^ neurons in AD [90], where PV^+^ cells are particularly relevant to disease [197–200]. Indeed, PNN loss is associated with neuronal death and/or degeneration in a number of disease contexts [160, 169, 176, 180, 190, 193, 201]. It should be noted that several studies failed to detect differences in PNN abundance in clinical AD [202–205], and this may be partly attributable to the brain region under investigation or the method of PNN labeling used. While changes with non-diseased aging are less clear [90, 206–208], we and others have found age-related reductions in PNN density in older animals [90, 193, 208, 209], and discrepancies across studies may depend on the inclusion of both sexes and the brain region(s), mouse strain, and/or model organism being studied.

**Fig. 1:**
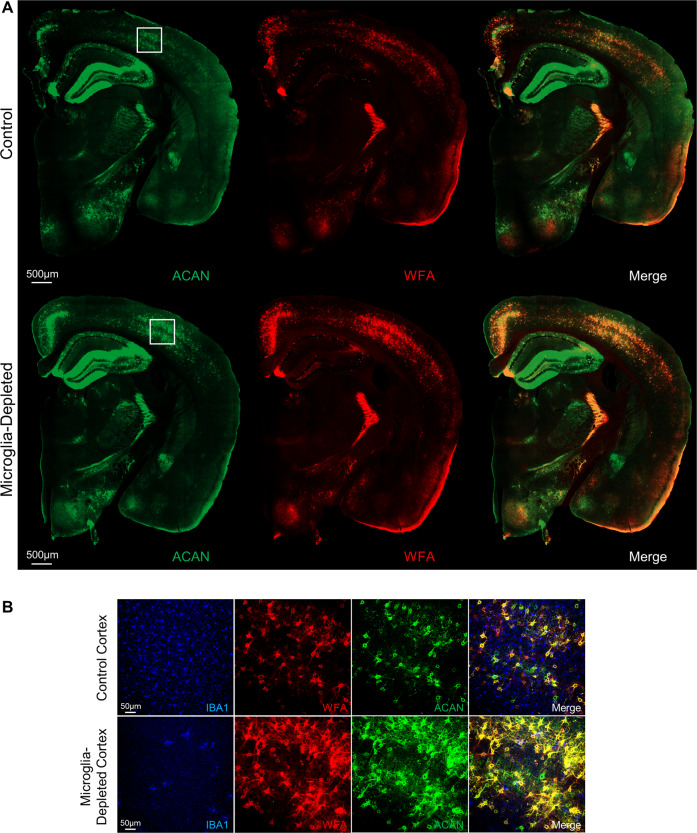
Microglial depletion enhances perineuronal net abundance in the healthy adult brain. Immunohistochemically stained brain sections from wild-type male mice aged 3 months that were treated with vehicle (Control) or the CSF1R inhibitor PLX5622 at 1200 ppm for 10 days (Microglia-depleted). Brain sections were stained with antibodies against aggrecan (ACAN; AB1031, Millipore) and with the canonical PNN marker *Wisteria floribunda* agglutinin (WFA; B-1355, Vector Labs). Effects are displayed as **A** whole-brain stitched images or **B** 20× confocal images of the somatosensory cortex from the same brain sections (white boxes in (**A**)) together with IBA1 to show microglial depletion

Even more striking, however, is the observation that PNN abundance is dramatically upregulated throughout the healthy adult brain following microglial depletion (Fig. 1) [72, 73, 90]. While neurons and glia may both express components that contribute to PNNs [94], neurons can express the core components of nets themselves and are capable of forming PNNs in vitro in the absence of glia [210]. For instance, neurons produce aggrecan [211], the inducible neuron-specific removal of which results in PNN ablation [147], as well as hyaluronan [212], which is continuously secreted and serves as a backbone by tethering associated CSPGs into PNNs with the aid of link proteins [213–215]. Therefore, our findings suggest that microglia basally regulate PNN density in the homeostatic brain, whether via direct or indirect enzymatic degradation and/or phagocytosis, such that their absence allows PNN components to accumulate. PNN enhancements induced by microglial depletion are also associated with increased excitatory and inhibitory synaptic connections to excitatory cortical neurons, as well as augmented neural activity in both cortical excitatory neurons and PV^+^ interneurons as assessed by in vivo calcium imaging [72]. Importantly, no overt changes in astrocytes were found in this study, as reported previously following pharmacological depletion in wild-type (WT) mice with either CSF1R inhibitor utilized [10, 71, 73]. Synaptic connectivity and neural activity are both normalized following microglial repopulation [72], which is consistent with the normalization of PNN densities we observed under similar conditions of inhibitor cessation following microglial elimination [216]. Loss of PNNs with disease thus likely reflects a toxic gain-of-function in microglia of this newly identified homeostatic role, whereby augmented or complementary PNN-degradative processes are activated, either via enhanced or alternative secretion of ECM-cleaving proteases or their modulators, and/or increased phagocytosis. Nonetheless, it remains possible that the effects reported here are mediated at least in part through associated downstream non-microglial pathways—such as astrocytes (e.g., via altered expression of gap junction channel subunit connexin 30, which facilitates PNN formation via inhibition of MMP9 expression [217]) or oligodendrocyte lineage cells [218], the population dynamics of which are altered by microglial depletion [55, 74]—and future studies should aim to resolve the distinct roles of glial and neuronal cells that may also be involved in PNN remodeling.

### Perisynaptic matrix

The vast majority (98%) of CSPGs within the CNS are found in the general diffuse ECM, including the perisynaptic matrix, as opposed to the highly specialized manifestations of the brain ECM that are PNNs [94]. Many key molecules coexist in both the perisynaptic matrix and in PNNs, so it is inherently difficult to tease apart the effects of their manipulations as resulting from changes to one or another ECM structure, particularly in the case of global genetic ECM knockout models. For instance, while aggrecan is a requisite component of the PNN backbone [147, 202, 219], the shorter CSPG brevican may be considered a reciprocally critical molecule to the perisynaptic ECM [165, 202, 215] and accumulates in synaptic fractions following biochemical fractionation of brain tissue [220, 221]. However, these CSPGs can be found across both structures, so additional research will likely be required to determine where and how they exert their effects on neuronal and synaptic physiology, and the extent to which interactions exist between ECM compartments. Interestingly, neuroglycan C (a.k.a. CSPG-5) appears to localize to perisynaptic regions of glutamatergic and GABAergic terminals and is often observed at the edges of PNNs [222], and its loss results in presynaptic functional deficits and premature elimination of synapses during development [223].

Several approaches exist to study the perisynaptic vs. perineuronal matrix. Mice deficient in link proteins HAPLN1 and HAPLN4, which serve to stabilize interactions between hyaluronan and CSPGs in PNNs [87], have overall unchanged CSPG levels but fail to incorporate these molecules into PNNs [94, 213, 224], and therefore may provide a means of studying the effects of disrupting these structures without affecting the perisynaptic matrix and diffuse ECM at large. Microinjections of ChABC near dendrites have also been successfully used to locally degrade perisynaptic CSPGs while leaving PNNs intact [225]. Alternatively, studies elucidating the function of the perisynaptic ECM could focus on regions that naturally lack PNNs [226], and vice versa in regions densely enriched with PNNs [227], although the effects of the perisynaptic matrix would not be entirely absent—just relatively minimized—in the latter case.

Research by several groups in the past decade has begun to shed light on the comparative composition of perisynaptic and PNN matrices in the CNS, as well as the organizational frameworks that distinguish them [161, 205, 215, 226–230]. The discovery of axonal coats (ACs) serves as one such example of a well-characterized perisynaptic matrix structure that exists as a separate entity from classical PNNs [230]. These round structures of aggrecan- and brevican-based ECM enwrap individual synaptic boutons contacting neuronal dendrites and somata and sometimes comingle with PNN components on associated neurons [215, 226, 230], with hypothesized roles at the synapse in restricting neurotransmitter spillover and receptor localization [87, 215, 227]. Although there is some degree of overlap, perisynaptic ECM can be found around neuronal subsets lacking PNNs [226, 230], as is the case for dopaminergic neurons and glutamatergic principal neurons in the substantia nigra and thalamus, respectively, on which presynaptic ACs make contact [230].

Additionally, activation of dopamine receptors and subsequent neuronal activity was shown in an elegant study to induce proteolysis of perisynaptic brevican and aggrecan in the ECM around excitatory synapses which, at least for brevican, was mediated by ADAMTS-4/5 [229]. It has also been shown that targeted perisynaptic matrix degradation induces structural plasticity of dendritic spines (e.g., enhanced spine motility and formation of spine head protrusions) [225] and similar structural changes are associated with increased functional plasticity as measured by LTP [231], and as such, CSPGs appear to restrict plasticity in either case. Thus, changes in upstream perisynaptic ECM could lead to downstream signaling-dependent changes in synaptic plasticity and further alterations in associated ECM in an increasingly complex, circuit-level process. Interestingly, we found elevated interstitial CSPG deposition in the brain parenchyma of both AD [90] and HD [73] mice, which may at least partially account for some of the beneficial effects of ChABC injections in related disease models that may have acted on the perisynaptic ECM rather than (or in addition to) PNNs [232–234].

**Fig. 2:**
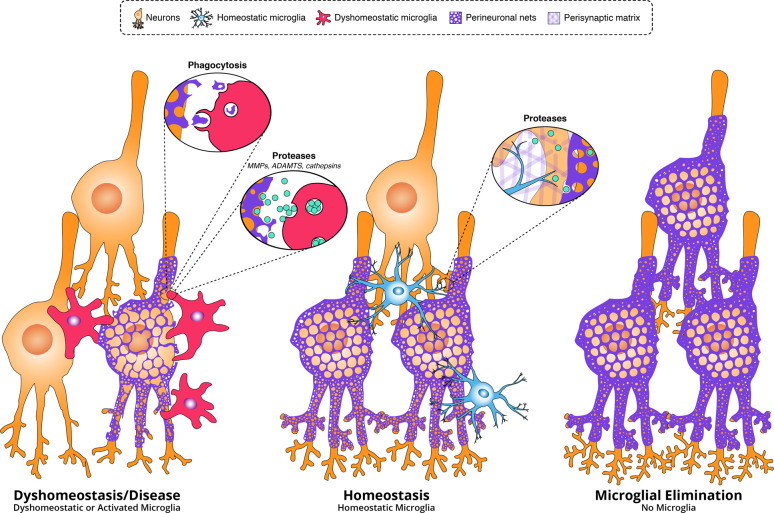
Microglia regulate perineuronal net and synaptic integrity in health and disease. In this working model, microglia continuously maintain baseline PNN and perisynaptic extracellular matrix integrity in the healthy adult brain through the sustained release of proteases (or protease inhibitors/activators) and/or phagocytosis (not pictured). The absence of local microglia through experimental depletion enhances PNN deposition and density, in addition to synaptic number. In disease or injury, microglial activation or dyshomeostasis leads to upregulation of phagocytosis and/or protease secretion, resulting in PNN breakdown and excessive synaptic elimination, the latter of which may occur through related and/or unrelated cellular pathways

Suggesting a direct role for microglia in the regulation of perisynaptic matrix-controlled synaptic plasticity, a recent study by Nguyen et al. determined that, in response to neuronal IL-33, microglia in the adult brain phagocytose and clear perisynaptic ECM components to promote dendritic spine formation, synaptic plasticity, and fear memory precision [91]. Importantly, they found that inhibition of this pathway decreased microglial engulfment of aggrecan and consequently enhanced aggrecan puncta density and deposition at the synapse, in addition to increasing total intact brevican while reducing levels of proteolyzed brevican. Thus, as in our work, loss of microglial function results in enhanced ECM deposition in the homeostatic brain. The occurrence of this phenomenon across multiple ECM compartments (i.e., the perisynaptic matrix [91] and perineuronal nets [73, 90]) together suggests a fundamental homeostatic role for microglia in ECM degradation and remodeling, which may be required for subsequent remodeling of synapses surrounded and stabilized by such ECM. These findings as they relate to PNNs are illustrated as a working model in Fig. 2. It should be noted that microglia may also act as a source of CSPGs and other ECM molecules in the interstitial matrix under certain conditions [139, 235], but this appears to represent a less prominent role compared to the negative regulatory influence they exert across ECM compartments.

### Potential mechanisms of microglial ECM regulation

Although microglia are linked to ECM remodeling in disease (i.e., PNN loss [73, 90, 166–169, 180, 181, 190]) and now also in the healthy homeostatic brain [72, 73, 90, 91], the molecular mechanism(s) by which this occurs are unclear. As observed with synapses during developmental pruning [37, 236], microglia may directly engulf and phagocytose ECM components. Indeed, aggrecan colocalizes with lysosomal CD68 in microglia, a marker of phagocytosis, and disrupting IL-33-based ECM engulfment by microglia reduces CD68^+^ lysosome number [91]. Furthermore, proteoglycans and PNN material can accumulate within disease-associated microglia/macrophages [90, 237] and in microglia following ketamine treatment [175], and phagocytic genes (e.g., *Itgax, Clec7a,* and *Trem2*) are upregulated in 5xFAD mice [67, 71], particularly in plaque-associated microglia [62], where we observed widespread PNN loss [90]. However, it is likely that microglial release of degradative enzymes is also involved in ECM turnover processes, especially as it applies to the remodeling of PNNs, in which CSPGs, tenascins, hyaluronan, and link proteins are more tightly woven together compared to the diffuse matrix [238].

Several proteases are immediately apparent candidates based on the capability of microglia to produce them and their ability, in turn, to degrade ECM components and core PNN molecules. These proteases primarily include MMPs, ADAMTS, and cathepsins. Microglia may also shape PNNs indirectly via modulators of protease activity, as in tissue inhibitors of metalloproteinases (TIMPs) [239–241], or by regulating protease or TIMP expression by other cells. For instance, it has been suggested [169] that glutamate released by activated microglia [242] could bind neuronal glutamate receptors and induce neuronal MMP expression [243]. While these are important and plausible mechanisms, for the purpose of conciseness, this review will focus on the direct action of microglia-sourced protease candidates.

MMPs are expressed at low to undetectable levels under homeostatic conditions in the adult brain and are upregulated in injury and disease; taken together, they have the capacity to degrade the entire gamut of ECM constituents [239]. Although not exclusively, MMP-2 and MMP-9 are secreted by microglia [244, 245] and act on a wide range of overlapping substrates, such as link proteins and aggrecan [239]. Substrate specificity is also evident in certain cases, as in the digestion of tenascin-C [246] and brevican [247] by MMP-2 but not MMP-9. MMP-2 and/or MMP-9 are upregulated by microglia in disorders where PNN breakdown occurs, such as stroke [248, 249], multiple sclerosis [250, 251], and glioma [252, 253], and pharmacological MMP blockade in glioma ameliorates enhanced MMP-2/9 activity and associated PNN loss [160]. While baseline PNNs are largely unchanged in MMP-9^−/−^ mice, developmental monocular deprivation-induced PNN degradation is prevented, and ocular dominance (OD) plasticity is attenuated [254], findings mirrored in adult mice in the context of light reintroduction-induced plasticity following dark exposure [255]. Additionally, genetic reduction (e.g., haploinsufficiency) of MMP-9 [256] as well as MMP-2/9 inhibitor treatment [257] restores developmental PNN impairments in *Fmr1* knockout mice, a model of Fragile X Syndrome (FXS); interestingly, MMP-2/9 inhibitor treatment also enhances WT PNN formation in the developing auditory cortex [257]. Of course, other MMPs may also play a role in brain ECM remodeling, as suggested by microglial *Mmp14* upregulation following treatment with IL-33, which endogenously promotes ECM clearance and dendritic spine formation [91]. Corroborating this, we also independently identified *Mmp14* upregulation in *Csf1r*^*+/−*^ mice, a model of leukoencephalopathy and microglial dyshomeostasis [172, 173], and confirmed its capacity to degrade PNNs via in vivo injection of recombinant MMP-14 (K.N.G., unpublished data).

In addition to MMPs, microglia can also express ADAMTS-4 [91, 258, 259], which cleaves aggrecan [260] and brevican [247] at sites distinct from MMPs and, unlike MMPs, degrades CSPGs without affecting laminin [261]. Furthermore, the effects of MMP-2 and ADAMTS-4 are additive in degrading brevican [247], which may offer one plausible explanation for the ability of exogenous ADAMTS-4 to degrade PNNs in amyotrophic lateral sclerosis model SOD1^G93A^ mice in which PNN breakdown had already occurred, but not WT mice [262, 263]. Microglial cathepsins also represent prime candidates in brain ECM turnover. Canonically localized to and functioning within the endolysosomal pathway to degrade proteins in bulk, several secreted cathepsins exist, including cathepsins S (CTSS) and B (CTSB) [244, 264]. CTSB is secreted by microglia following LPS activation [265], as is CTSS, which is also upregulated by brain lesion injury [266] and in bulk tissue of 5xFAD mice where we have reported PNN deficits (hippocampus, cortex) [71, 90]; it should be noted in the latter case that we did not observe significant upregulation of any *Mmp* genes in any regions examined [71]. We found that *Ctss* expression in the brain most closely follows the kinetics of microglial elimination and repopulation [74], which increases [72, 73, 90] and normalizes [216] PNN density, respectively, and indeed, its transcripts were consistently absent in microglia-depleted brains in our studies [71, 73, 74, 216]. CTSS is functionally stable at the neutral pH of the extracellular space, and under such conditions, it can efficiently degrade CSPGs neurocan and phosphacan, where CTSB at several-fold greater concentrations could not [266]. Further supporting the plausibility of CTSS-based ECM remodeling in particular, CTSS^−/−^ mice display ameliorated tenascin-R reduction following facial nerve axotomy [267], which induces CTSS (but not CTSB) upregulation at the protein and mRNA levels, and incubation of mouse brain sections with CTSS eliminates WFA^+^ PNNs [268].

On the other hand, the upstream signals that trigger the clearance of ECM by microglia, whether by protease secretion and/or phagocytosis, and whether this differs across ECM compartments, together remain largely unknown. Neuronal IL-33 guides the engulfment of perisynaptic ECM by microglia in the homeostatic adult hippocampus [91], but it is unclear whether this pathway also guides the microglial regulation of PNNs. Experimental designs focused on the latter may benefit from targeting the expression of IL-33 or other candidate signaling molecules in PV^+^ interneurons (e.g., through a Cre-lox system), given the close association between this neuronal subtype and PNNs. As microglia appear to basally regulate PNN abundance, with their depletion enhancing PNN densities in the healthy brain [72, 73, 90], the signal(s) regulating this process should theoretically be homeostatically secreted. The CX3CL1-CX3CR1 axis is already well established as a major pathway of neuron-microglia communication that is involved in synaptic pruning and development [38, 269, 270] as well as microglial mobility, motility, and activation [270–272] and therefore seems to be a feasible candidate in the regulation of this process. However, PV^+^ neuron-associated PNN densities remain unchanged in *Cx3cr1*^*−/−*^ mice [273], and thus, this pathway appears to be uninvolved in PNN remodeling by microglia.

The homeostatic microglial receptor P2RY12 may instead serve to regulate microglial-PNN interactions, as blocking P2RY12 with the specific antagonist clopidogrel prevents ketamine-induced loss of PNNs in adult mice [175] and inhibits developmental ocular dominance plasticity [274], which is thought to be typically restricted by the formation of PNNs [146]; therefore, future studies may benefit from evaluating the functionality of P2RY12 in this regard. CSF-1/IL-34 could also represent putative regulatory molecules, as they are constantly produced in the brain at baseline and control microglial survival and cell densities by signaling through CSF1R [275]. We found that *Csf1r* haploinsufficiency (*Csf1r*^*+/−*^), as well as low-grade, brain-penetrant pharmacological CSF1R inhibition (150 ppm PLX5622), induced PNN deficits, which were rescued following microglial depletion with high doses of CSF1R inhibitors (Arreola et al., *in press*). Furthermore, alternative or complementary signaling pathways may be involved in the regulation of PNNs by microglia under conditions of dyshomeostasis and disease (e.g., via the detection of DAMPs by microglial TLRs [252]) which may, in turn, vary based on disease etiology and pathogenesis. Toxic gain- or loss-of-function in homeostatic signaling pathways regulating microglia-PNN interactions in the healthy brain may also occur in disease. Ultimately, the role of microglia as sculptors of PNNs and the ECM in general—particularly in the healthy brain—is just beginning to be elucidated, and as such, the signals promoting this process, as well as the downstream mechanisms mediating such sculpting, require further study.

The proteases proposed in this section are implicated in remodeling not only the ECM, but synapses as well [264, 276, 277]. This may underscore the functional relationship between the two structures—to sculpt synapses, an increasingly salient role of microglia, the matrix in which they are embedded would presumably also have to be restructured. Ongoing research continues to elucidate the bidirectional interactions between the ECM and synapses, but the involvement of microglia in this process has just begun to be examined [91]. Therefore, we will next discuss established findings on synaptic regulation by microglia in the context of specific and relevant ECM studies to shed light on putative mechanisms that may underlie the relationship between these components of the tetrapartite synapse.

## Microglia at the synapse

### Synaptic pruning and formation in development

Thorough monitoring of the CNS parenchyma by microglia [13] aptly positions these cells to respond rapidly to changes in the synaptic microenvironment. In the healthy brain, they interact with pre- and postsynaptic compartments, perisynaptic astrocytes, and the local extracellular milieu [88, 89, 278, 279]. This has thus far been best studied during development when microglia prune excess synapses [37, 38] to promote the removal of extranumerous or weak synapses in the refinement of neuronal networks [21, 35]. Accumulating evidence has implicated traditionally immune-associated molecules as critical elements in synaptic refinement. For example, complement cascade elements (e.g., C1q and C3) localize to synaptic compartments to tag synapses for elimination [39, 280, 281], inducing phagocytosis by complement receptor 3 (CR3)-expressing microglia in a neural activity-dependent manner [37]. On the other hand, genetic loss of CX3CR1, a receptor primarily expressed by microglia in the brain, is also associated with synaptic pruning deficits, resulting in an excess of dendritic spines, immature synapses, and immature brain circuitry in development [38, 269, 270] that persists as impaired synaptic transmission and functional brain connectivity in adults [64].

Microglia can also induce synapse formation, as shown by the addition of developing microglia to cultured hippocampal neurons in vitro, which increases dendritic spines and excitatory and inhibitory synapses via microglial IL-10 [282]. While this process did not require direct microglial contact, a recent study utilizing in vivo two-photon imaging of early postnatal (P8-P10) mouse brains observed microglial contact-induced filopodia formation on dendrites, which was reduced following minocycline treatment [16]. Decreased dendritic spine densities were observed in the same study following microglial depletion [16], which resembled the reduced spine formation reported by another group under similar circumstances [283]. However, caution must be taken regarding the interpretation of this result, as both studies utilized diphtheria toxin-based models of microglial ablation, which are associated with inflammation (e.g., upregulation of TNF-ɑ, IL-1β [8] or an interferon response [284]) that is not seen with genetic- or inhibitor-based models due to the manner in which microglial death is achieved [11]. Accordingly, IL-1β attenuates synaptic formation induced by IL-10 [282], and postnatal CSF1R inhibitor-based microglial depletion instead results in excess synapses [285] that are normalized following microglial repopulation [286]. Interestingly, loss of CSPG-5 (neuroglycan C), which normally localizes to the perisynaptic space [222], results in impaired presynaptic maturation as well as synaptic elimination that occurs earlier than normal in cerebellar Purkinje cells [223], which microglia survey and regulate [287–290]. As early developmental synaptic deficits are observed in other brain regions with CSPG-5 deficiency [291], together, this suggests a role for perisynaptic matrix remodeling during synaptic pruning and maturation.

It is perhaps no coincidence that PNNs begin forming in development soon after synaptic pruning is completed by microglia [38, 39], ~P14 in mouse cortex (finished by P40) and earlier in subcortical regions [213, 292], which would place them in ideal positions to guide PNN formation around newly refined synapses, e.g., through phagocytosis and/or controlled enzymatic degradation. MMP-2/9 inhibitor treatment enhances basal PNN density in postnatal mice, indicating that protease activity is indeed a limiting factor in their developmental construction [257]. Of note, MMP-2 and MMP-9 expression peaks in early postnatal development, where they codistribute with foci of proteolytic activity in neuropil, and with markers of synapses (PSD-95, synaptophysin) and growing axons, suggesting that these proteases actively shape the perisynaptic space associated with synapse formation [293]. Furthermore, the formation of adult levels of PNNs around visual cortical neurons by the end of the critical period restricts OD plasticity [146], such that their ablation in adults restores OD plasticity [146, 147, 149, 213], and the loss of microglial P2RY12 [274] (but not CX3CR1 [294]) or microglia themselves with CSF1R inhibition [295] prevents normal OD plasticity altogether.

Therefore, we postulate that the absence of OD plasticity following such microglial loss-of-function may be due to consequent failure to sculpt PNNs, which may form prematurely in these instances. Interestingly, synaptic elimination in the barrel cortex following developmental whisker trimming—which also specifically reduces barrel cortex PNNs [296]—requires CX3CR1 [297], while neither CX3CR1 [294] nor C1q [298] appear necessary for monocular deprivation-induced OD plasticity or related visual cortex synaptic remodeling. Thus, microglial mechanisms of synaptic sculpting are context-dependent, and this may also be the case for the regulation of nearby ECM. Future studies should investigate to what extent microglia and the microglial proteome are involved in regulating PNN formation during critical period closure and how this may relate to synaptic pruning. This could be explored via developmental or critical period CSF1R inhibition to determine whether PNNs appear earlier, and further delineated with a more specific approach (e.g., protease inhibitors) to determine exactly how microglia influence this process.

The pursuit of clarification regarding mechanisms that dictate which synapses are eliminated or spared has uncovered a delicate balance between ‘eat me’ and ‘do not eat me’ signals at the neuronal level. Phosphatidylserine (PS) localized to synaptic elements is one such molecule by which the CNS can modulate synaptic elimination [299, 300]. While initially recognized as a glycerophospholipid that is externalized on the cell membrane during the process of apoptosis to act as an ‘eat-me’ signal for phagocytes [301], synaptic elimination is partially abrogated in vitro by blocking PS via the addition of Annexin V or by culturing with microglia deficient for the phagocytic receptor TREM2 [300]. In vivo, synaptic PS exposure in the hippocampus and retinogeniculate areas parallels the temporal dynamics of microglial-mediated pruning, and C1q-deficient mice displayed increases in presynaptic PS exposure and reductions in PS phagocytosis by microglia, thereby implicating the complement system in PS-mediated synaptic pruning [300]. Aside from complement-related mechanisms, microglial loss of GPR56 decreased engulfment of PS^+^ synaptic inputs and consequently increased synapse number in the hippocampus and dorsal lateral geniculate nucleus [299], suggesting that multiple signaling pathways involved in the regulation of microglial pruning appear to converge on synaptic PS expression.

On the other hand, a recent study identified that CD47 signaling to the SIRPα receptor serves as a “do not eat me” signal that prevents excessive synaptic pruning in the retinogeniculate system during early development [236]. CD47 is enriched, and microglial expression of SIRPɑ is similarly increased during peak pruning, with CD47 localizing to more active synapses, and disruptions to either via knockout of CD47 or SIRPα increased microglial engulfment and reduced synapse number [236]. Studies such as these provide insight into the relationship between microglia and synaptic structures and, importantly, describe how the developing nervous system can exert spatiotemporal control over synapse elimination. Along these lines, a subpopulation of GABA-receptive microglia has recently been identified that specifically prunes inhibitory synapses in development [302]. Importantly, ablating the microglial GABA_B_ receptor subunit GABA_B1_R to disrupt GABA_B_ signaling in microglia, which mediates this effect, did not alter PNN densities [302]. Additionally, recent proteomic studies have identified a number of putative MMP-9 substrates, including nuclear, cytoplasmic, and extracellular proteins not solely involved in the ECM but that may have implications in synaptic plasticity, including Annexin V [303]. Intriguingly, it appears that MMP-based proteolytic cleavage of SIRPα in response to neuronal activity releases an extracellular SIRPα domain, which binds to presynaptic CD47 and promotes the maturation of presynaptic terminals [304]. ECM molecules may also interact with synaptic pruning pathway elements, as CSPGs bind and potentially inhibit C1q functional domains [305, 306], whereas loss of CD47 in glioblastoma cells enhances the expression of tenascin-C and consequent phagocytosis by tumor-associated macrophages [307].

While the prevailing theory is that microglia phagocytose whole synapses, recent advances in microscopy are facilitating greater direct imaging of microglial interactions with synapses at resolutions that may reveal more nuanced roles in synaptic remodeling. Such techniques have allowed researchers to observe microglia contacting presynaptic elements in hippocampal explants and subsequently phagocytose only fragments of the synapse in a process termed presynaptic trogocytosis [308], a phenomenon recently confirmed in vivo in *Xenopus laevis* tadpoles [309]. However, while Weinhard et al. found that complement signaling (specifically CR3-mediated) was not required for trogocytosis [308] as it is in developmental retinogeniculate pruning [37], complement signaling did regulate trogocytosis of retinal ganglion cell axons in *Xenopus laevis* [309]. In the latter case, neuronal overexpression of the synapse-associated amphibian regulator of complement activation 3 (aRCA3) inhibited trogocytosis and axonal pruning, whereas expression of axon membrane-bound complement C3 fusion protein enhanced axonal pruning [309]. Placing this in the context of the ECM, several studies suggest that perisynaptic axonal coats are synthesized by presynaptic neurons [205, 215, 226, 230] and thus may have particular relevance in the microglial trogocytosis of presynaptic components [308] in that this process might necessarily involve concurrent remodeling of the presynaptically generated ECM that supports synapses in these instances as well as a mechanism in place to allow such preferential targeting by microglia.

### Synaptic elimination in the healthy adult brain and its dysregulation in disease

Although largely studied in the context of development thus far, growing research indicates that microglia maintain their roles as synaptic sculptors of the adult homeostatic brain, a function that may go awry in disease. Supporting this, we have demonstrated that elimination of microglia in healthy adult mice with CSF1R inhibitors increases the total density of hippocampal and visual cortex dendritic spines and PSD95 and synaptophysin immunolabeling in the hippocampus [66, 76]. Similarly, microglial elimination increases excitatory and inhibitory connections to visual cortex excitatory neurons and the neural activity of excitatory neurons and PV^+^ interneurons [72], in addition to enhancing PNN density [72, 73, 90], confirming that microglia serve as regulators of the synaptic and ECM landscape throughout adulthood. Indeed, as in development [37], hippocampal microglia continue to perform activity- and complement-dependent synaptic elimination to mediate normal memory turnover in the healthy adult brain, such that complement inhibition or microglial depletion prevents forgetting of contextual fear memories [40].

Neurogenic niches in the adult CNS, such as the olfactory bulb (OB) and dentate gyrus (DG) of the hippocampus, provide unique perspectives on synaptic modulation by microglia, as new neurons are continuously born, develop, and integrate into functional neuronal circuits in an already mature brain environment. Accordingly, elimination of microglia via CSF1R inhibition is reported to reduce the spine density of developing but not mature adult-born granule cells (abGCs), suggesting that microglia are necessary for the proper development of synapses in adult-born neurons [310]. This is mediated in part by CX3CR1 [310], as paralleled by a separate report on impaired synaptic integration and reduced spine density at the afferent level in adult-born granule neurons of the hippocampal DG in *Cx3cr1*^−/−^ mice [273]. Another group found that microglial depletion with the same CSF1R inhibitor resulted in enhanced spine density on developing abGCs, but these spines were smaller and functionally immature; again, the effects were largely limited to young but not mature abGCs [311]. Taken together, these data suggest that the requirement of microglial CX3CR1 for the synaptic refinement of adult-born neurons in the OB [310] and DG [273] is a necessity only in their developmental stages, which could be determined with the use of inducible rather than constitutively deficient *Cx3cr1*^−/−^ mice.

Interestingly, the absence of CX3CR1-mediated bidirectional communication between microglia and neurons in *Cx3cr1*^−/−^ mice was also sufficient to enhance WFA^+^ and aggrecan deposition in the DG, where synaptic integration was impaired [273]. However, the authors found no difference in PNN density here, and the elevated proinflammatory profile of this region (e.g., TNF-ɑ, IL-6) [273] suggests that these changes could primarily be localized to the diffuse ECM via microglial activation of neurotoxic astrocytes [45] and their increased production of neurite-inhibitory CSPGs in turn [114]. In fact, activation of primary cortical microglia with polyinosinic-polycytidylic acid in vitro induced secretion of TNF-ɑ and IL-6 in the culture medium, in addition to several chemokines, and upregulated expression of *Mmp2* and *Mmp9*, and treatment of hippocampal neurons with this microglial-conditioned medium impaired PNN structure [312]. Treatment of PNN-ensheathed neurons with this medium also led to a decrease in inhibitory vGAT presynaptic puncta but an increase in PSD-95 and gephyrin postsynaptic markers, whereas both inhibitory vGAT and excitatory vGlut presynaptic puncta were reduced while postsynaptic markers were unaffected in treated non-PNN-ensheathed neurons, underscoring the unique and complex role of PNNs in scaffolding and regulating embedded synapses [312]. In line with this complexity, others have reported that PNN disruption by genetic deletion of its components in primary hippocampal neurons in vitro transiently increases synaptic densities, only to later reduce them [164]. In vivo, the situation is likely different if not more complicated, as microglia can make direct contact with PNNs and synapses, potentially remodeling these structures via phagocytosis [40] as well as through secretion of proteolytic factors.

While synaptic elimination is known to be a normal process in brain development and homeostasis, the dysregulation of this process is recognized as an early feature of neurodegeneration [313–315]. Synaptic loss, as opposed to neuronal loss, serves as the most accurate indicator of cognitive decline [313, 316, 317]. Under neurodegenerative conditions, microglial-induced synapse loss may be viewed as a toxic gain-of-function with respect to normal synaptic-regulating processes [318], as in models of AD, where dysfunctional activation and upregulation of complement proteins C1q and C3 [319–321] or loss of microglial SIRPɑ [322] result in excessive phagocytosis of synaptic elements. Augmented complement-mediated synaptic loss also appears to occur in aging [323]. Microglia can also increase the expression of synaptotoxic factors such as TNF-ɑ in neurodegeneration, which produces synaptic deficits by inducing excitotoxicity [324, 325] or by promoting neurotoxic astrocyte reactivity [45]. Elimination of microglia or attenuation of microglial activation under neurodegenerative conditions or aging, however, leads to improved functional outcomes accompanied by restoration in spine number and synaptic surrogates [66, 67, 71, 77, 326, 327]. Thus, microglia play critical roles in the maintenance and pathological elimination of synaptic elements in disease (as reviewed previously [328]).

Few studies have explored the roles of microglia in the ECM as they relate to changes in synaptic health and number in neurodegenerative contexts. Microglial depletion prevents PNN loss in the 5xFAD hippocampus [90] and the downregulation of hippocampal synaptic genes at later time points [71], but further investigation is required to clarify whether these changes  are occuring in the same neurons. Neurodevelopmental disorders may provide another avenue to investigate such processes, as in FXS, which is caused by genetic hypermethylation-induced loss of neurite-localized fragile X mental retardation protein (FMRP) [329]. Minocycline-based inhibition of MMP9 [330] or *Mmp9* deficiency [331] rescues the immature dendritic spine phenotype in fragile X mouse hippocampal neurons in vitro, and pharmacological blockade [257] or genetic reduction [256] of MMP-9 restores in vivo cortical PNN density in FXS mice, as discussed earlier. Future studies should investigate how these ECM and synaptic effects may be related in neurons from the same brain region and under the same experimental conditions. Similarly, anomalous synaptic deficits are postulated to be important components of pathology associated with schizophrenia, as reduced dendritic spine densities [332–334] and disrupted PNNs in similar cortical regions (e.g., layer 3 of the prefrontal cortex [195]) have been reported, in addition to aberrant microglial elimination of synapses in schizophrenia patient-derived neural cultures [335] that is related in part to disease variants in complement component 4 (C4) [335, 336]. Given the increasingly reported roles microglia appear to play in ECM modulation, both at the level of PNNs and the perisynaptic matrix, it stands to reason that such synaptic, ECM, and microglial changes may be related in these situations as well. Overall, studies into neurodegenerative and neurodevelopmental disorders are increasingly alluding to complex interactions between glia and other elements of the tetrapartite synapse that may be determined to underlie major aspects of disease pathophysiology.

## Concluding remarks

Altogether, the data thus far suggest that microglia serve a regulatory role in the modification of ECM and synaptic components; the goal now is to elucidate the dynamics of this relationship. Specifically, future studies should investigate how microglia mediate such ECM modifications (whether through protease secretion, phagocytosis, or a combination of both), how this process is resolved at the perisynaptic vs. PNN level, and how such alterations interface with synaptic function in CNS development, health, and disease. In terms of proteases, we propose MMPs, ADAMTS, and/or cathepsins as the most feasible mechanistic candidates given the current data, whether these molecules are directly expressed by microglia or are instead influenced by the secretion of other microglial factors in a more indirect manner.

At a more general level, it will be interesting to determine the extent to which PNN deficits are a common hallmark of neurodegenerative diseases, and furthermore how this relates to differential microglial phenotypes. It is also possible that some of the deleterious effects of diseased microglia on PNN integrity are mediated, if even only in part, by other glia (e.g., astrocytes [217] or oligodendrocytes [218]), which are often dysregulated concurrent with microglial dysfunction. However, the minimal changes in astrocytes evoked by microglial depletion in the homeostatic brain—particularly in comparison to the consequent dramatic and relatively ubiquitous upregulation of PNNs—together with the collective findings reviewed here suggest a central role for microglia in ECM and synaptic regulation. Thus, a novel role for microglia emerges in the basal regulation of PNNs and ECM in the healthy adult brain, and as with other microglial functions, this may serve as a valuable therapeutic target if, or when, it is pathogenically altered in disease.
